# Virological failure on first-line antiretroviral therapy; associated factors and a pragmatic approach for switching to second line therapy–evidence from a prospective cohort study in rural South-Western Uganda, 2004-2011

**DOI:** 10.11604/pamj.2018.29.191.11940

**Published:** 2018-04-02

**Authors:** Patrick Kazooba, Billy Nsubuga Mayanja, Jonathan Levin, Ben Masiira, Pontiano Kaleebu

**Affiliations:** 1 MRC/UVRI Uganda Research Unit on AIDS, Entebbe, Uganda; 2 Department of Clinical Research, London School of Hygiene and Tropical Medicine, Keppel Street, London

**Keywords:** First line ART, second line ART, virological failure, conventional, pragmatic

## Abstract

**Introduction:**

We investigated factors affecting Virological failure (VF) on first line Antiretroviral Therapy (ART) and evaluated a pragmatic approach to switching to second line ART.

**Methods:**

Between 2004 and 2011, we assessed adults taking ART. After 6 months or more on ART, participants with VL >1000 copies/ml or two successive VL > 400 copies/ml (Conventional VF) received intensified adherence counselling and continued on first-line ART for 6 more months, after which participants who still had VL > 1000 copies/ml (Pragmatic VF) were switched to second line ART. VF rates were calculated and predictors of failure were found by fitting logistic regression and Cox proportional hazards models.

**Results:**

The 316 participants accrued 1036 person years at risk (pyar), 84 (26.6%) had conventional VF (rate 8.6 per 100 pyar) of whom 28 (33.3%) had pragmatic VF (rate 2.7 per 100 pyar). Independent predictors of conventional VF were; alcohol consumption, (adjusted Hazard Ratio; aHR = 1.71, 95% CI 1.05-2.79, P = 0.03) and ART adherence: per 10% decrease in proportion of adherent visits, (aHR = 1.83, 95% CI 1.50-2.23; P < 0.001). Using reference age group < 30 years, among conventional failures, the adjusted odds ratio (aOR) of pragmatic failure for age group 30-39 years were 0.12, 95% CI 0.03-0.57, P = 0.02 and for age group > 40 years were 0.14, 95%CI 0.03-0.71, P = 0.02. Alcohol consumers had a threefold odds of pragmatic failure than non-alcohol consumers (aOR = 3.14, 95%CI 0.95-10.34, P = 0.06).

**Conclusion:**

A pragmatic VF approach is essential to guide switching to second line ART. Patient tailored ART adherence counselling among young patients and alcohol users is recommended.

## Introduction

Antiretroviral therapy (ART) has changed the natural history of HIV infection [1]. By the end of 2015 about 36.7 million individuals were living with HIV globally and 25.8 million of these were living in sub-Saharan Africa (SSA) [2, 3]. By June 2016, ART coverage had increased to 46% globally and 54% in SSA [2]. Uganda's HIV prevalence by 2014 was estimated at 7.4% and out of the estimated 1.5 million people living with HIV-AIDS (PLWHA)by the end of 2014, 0.75 million people were on ART and about 10% of these had HIV viral load of above 1000 copies/ml [4]. ART initiation reduces HIV replication in peripheral blood [5-8] suppresses plasma HIV viral loads (VL) to unquantifiable levels within 4-6 months [9, 10], reduces morbidity and mortality, with resultant improvement in survival [1, 5]. If adequate viral suppression is not achieved, therapy is considered to be failing and may require switching to a second line ART regimen [11, 12] which may be both expensive and toxic. Like other African countries, Uganda is rolling out HIV viral load testing which is expected to increase detection of individuals with virological failure who may need second line ART regimen [13]. HIV RNA virological monitoring is the gold standard for measuring ART progress [14-20]. Although HIV viral suppression requires good ART adherence in excess of 95% [21], suboptimal viral suppression due to poor adherence has undermined HIV care in SSA since introduction of free ART programmes in 2004-2005. Many other factors are independently associated with failure to achieve optimal HIV viral suppression [19]. Knowledge of such predictors will enable clinicians forecast ART outcomes and design interventions to prevent virological failure and meet the UNAIDS 90-90-90 treatment targets. Diagnosis of virological failure based on a single plasma VL measurement of > 1000 copies/ml or two successive VL measurements above 400 copies/ml, at any time after 6 months on ART, (conventional VF), has led to unnecessary switching from first to second line ART regimen which is costly and almost currently the last available treatment option in most SSA countries [22]. If patients with detectable VL after 6 months on ART receive further intensified ART adherence counselling and continue on first-line ART for another 6 months, only those whose VL remains >1000 copies/ml (true or pragmatic virological failure) are then switched to second-line ART. Until HIV drug resistance testing is widely available in public HIV care settings, identifying patients with true or pragmatic virological failure provides a more reliable criterion for switching patients failing on first line ART to second line ART. Studies in developed countries have evaluated switch strategies as a basis of enhancing cost savings in an era of global economic recession [23]. In this study, we assessed factors affecting virological failure using both the conventional and pragmatic definitions and justified the use of pragmatically defined virological failure while switching patients to second line ART. The study findings add to the body of knowledge on ART switching practices in resource limited settings and enhances prediction of virological failure risk so as to improve ART treatment outcomes.

## Methods

**Study design and setting**: The study was based in an open prospective HIV Rural Clinical Cohort (RCC) in southwest Uganda. The RCC was established in 1990 to study the natural history of HIV-1 disease progression and started providing ART and cotrimoxazole prophylaxis to eligible participants in 2004 according to the existing National Guidelines [24, 25]. First line ART consisted of Zidovudine, Lamivudine and either Nevirapine or Efavirenz. Until March 2008, patients who were found to be anaemic before or after initiation on Zidovudine were switched to Stavudine. In Uganda, Stavudine was thereafter gradually replaced by Tenofovir due to its toxicity. Second line ART was a combination of Tenofovir, Emtricitabine (or Lamivudine from July 2010) and ritonavir boosted Lopinavir. At the monthly ART refills, an adherence nurse assessed and documented ART and cotrimoxazole prophylaxis self-reported adherence and did a pill count. Every quarter, study clinicians conducted routine questionnaire interviews, collected medical and sexual behaviour history data, and did clinical examination and laboratory investigations. Free treatment was provided according to the National treatment guidelines [26]. From 2004 to 2011, PLWHA individuals aged 13 years and above who initiated and had been on ART for 6 months or more with at least 2 plasma viral load measurements including one at baseline were included in this analysis.

**Laboratory methods**: CD4 cell counts were measured at ART initiation and quarterly using the FACS Count machine (Becton Dickinson, San Jose, California, USA). Viral loads were measured at ART initiation and 6 monthly, between January 2004 and September 2007 we used the VERSANT RNA 3.0 (Bayer, Bayer Healthcare, NY, USA) assay (lower detection limit of 50 copies per ml), while from October 2007 to December 2010 the Amplicor MONITOR 1.5 (Roche, Roche molecular systems, NJ, USA) assay was used (lower detection limit of 400 copies per ml).

### Data management and statistical analysis

**Software and participant follow up**: Data was managed using an Ms Access database and was analysed in Stata version 13.0. Participants' characteristics at ART initiation between January 2004 and March 2011 were summarised by cross tabulation. The study had outcomes based on 2 virological failure definitions (conventional and pragmatic) at different time points. A participant's follow-up started at ART initiation and ended at the earliest time of diagnosis of virological failure, death, loss to follow up, or date last seen (whichever was first). Loss to follow up was defined as being late for 3 months or more after the next scheduled quarterly visit and still known to be alive. Participants were censored at the end of the study.

**Ethics and consent**: this study was approved by the Science and Ethics Committee of the Uganda Virus Research Institute and the Uganda National Council for Science and Technology. Participants gave signed or thumb-printed written informed consent to participate in the study and confidentiality procedures were adhered to throughout the study

**Outcome**: The main outcome was virological failure. Conventional virological failure was defined as a single VL of > 1000 copies/ml or two successive VL > 400 copies/ml taken 6 months apart from a participant on ART for 6 months or more. Participants with conventional virological failure received intensified adherence counselling and continued on first line ART for 6 more months after which those with persistent VL > 1000 copies/ml were regarded to be true or Pragmatic failures and all were switched to second line ART regimen.

**Exposures**: The main exposure was ART adherence which was measured monthly at each ART refill visit using both self-report and pill count. Participants reported the number of days they missed taking their doses over the previous four days and since the last ART refill visit. Adherence was categorised as: good (> 95%) if no doses were skipped over the last 4 days or since the last refill visit and poor (<95%) if any doses were missed over the same period. ART adherence data for each participant over the entire follow up period or up to the visit of virological failure was collapsed to create an overall adherence summary measure. The proportion of visits with good adherence was presented for each participant as categorical for crude analysis. In addition, the continuous variable (proportion of visits with good adherence) was rescaled so that 1 unit represented a 10% decrease in the proportion of visits with good adherence (e.g. from 100% to 90%) and this was used in the regression model. Secondary exposures included characteristics measured at ART initiation including gender, age, marital status, number of partners, occupation, alcohol consumption, CD4 counts, HIV viral loads, weight and body mass index (BMI).

**Figure 1:**
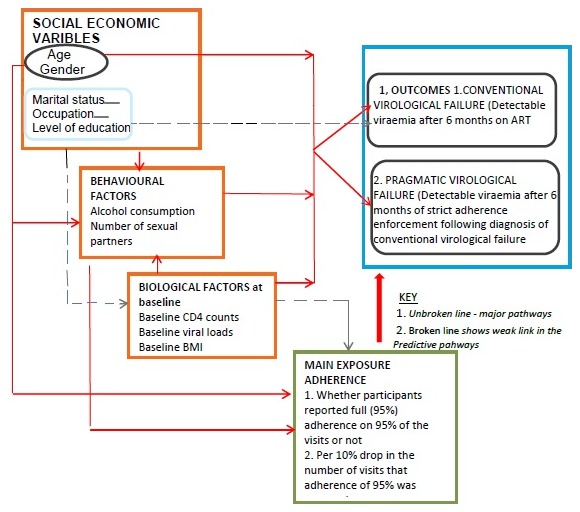
Conceptual framework for factors affecting virological failure and a pragmatic approach to diagnosis of virological failure

**Conceptual framework**: Our analysis was based on a conceptual framework which assumed; ART adherence was independently associated with virological failure. Other confounders included; socio-demographic (age, gender, marital status, occupation and level of education) behavioural (alcohol consumption and number of sexual partners) and biological (body mass index, baseline CD4 counts and baseline VL) risk factors. The association with ART adherence was considered distal for apriori confounders age and gender and proximal for alcohol consumption. Biological factors such as, high baseline VL was associated with low baseline CD4 counts, poor health, low BMI and poor ART adherence. We also assumed that, a high baseline VL provides a potential for ART drug resistance mutations which may lead to virological failure [27] (Figure 1).

**Virological failure rates by categories of explanatory variables**: Conventional and pragmatic virological failure rates were calculated and tabulated for categories of explanatory variables using standard survival analysis methods. Kaplan-Meier methods were used to estimate time to conventional and pragmatic virological failure. The crude hazard ratios and 95% confidence intervals (CI) were obtained using Cox regression analysis. The P-values for the hazard ratios were calculated from the likelihood ratio test (LRT).

**The effect of adherence on virological failure**: Cox regression models were used to estimate adjusted hazard ratios (aHR) and 95% CIs for the effect of adherence on conventional and pragmatic virological failure separately. To adjust for confounding, a multivariable model was built basing on the conceptual framework. A model was fit with the main exposure of interest, adherence, and apriori confounders (gender, age and alcohol consumption) and the hazard ratio for the exposure of interest was noted. Apriori confounders were selected basing on existing literature on their potential to affect adherence. Other potential confounders were added one at a time and the effect on the hazard ratio of the main exposure was noted. Potential confounding was considered if a variable changed the hazard ratio by 10% or more. Collinearity was considered if a confounder changed the log10 standard error of the main exposure of interest.

**Independent predictors of conventional virological failure**: A model to determine the independent predictors of conventional virological failure was based on a conceptual framework. Age, alcohol consumption and gender were left in the model as apriori confounders and other potential predictors were added one by one starting with those considered proximal (Biological) and ending with those considered distal in the conceptual framework. Factors were only left in the model if the P-value from the LRT was < 0.10. Independent predictors of conventional virological failure were analysed separately and statistical significance was assessed using LRT.

**Factors associated with pragmatic failure among the conventional failures**: The proportion of conventional failures that were also pragmatic failures for each level of the explanatory variables was calculated separately and logistic regression models were fitted to determine factors associated with being a pragmatic failure among the conventional failures. The odds ratios and 95% CI were estimated and statistical significance was assessed using LRT.

## Results

**Figure 2:**
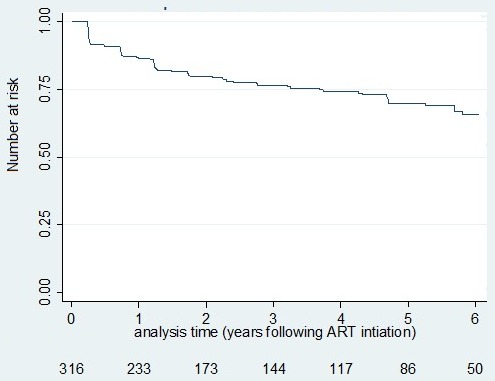
Conventional failure survival experience of HIV infected participants since ART initiation–Kaplan Meir estimate

**Figure 3:**
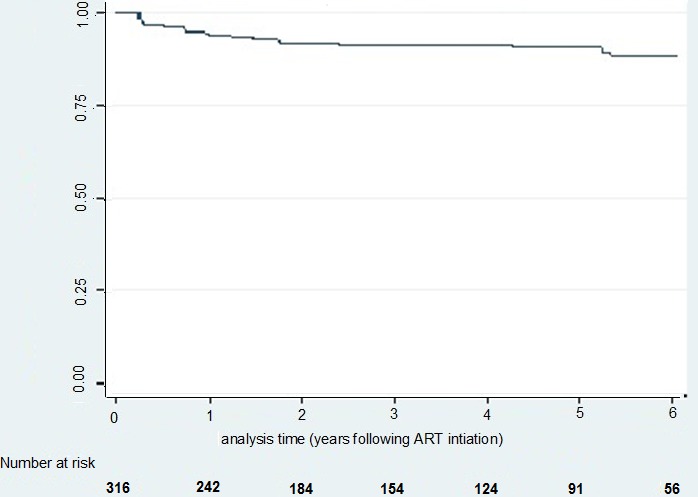
Pragmatic failure survival experience of HIV infected participants since ART initiation–Kaplan Meir estimate

**Participant's characteristics at ART initiation**: A total of 316 participants were initiated on first line ART from January 2004 to December 2011, 63.6% were females, 67.4% had no or incomplete primary education, 60.8% were farmers and 29.4% consumed alcohol. The median age at ART initiation was 36.5 years with 62.0% aged below 40 years, range 15 to 75 years. Participants initiated ART at a median CD4 count of 156 cells/μl (IQR 73-200) and a median VL of 81,758 copies/ml (IQR 26,721-193,184). The mean BMI at ART initiation was 20.1kg/m^2^ (Table 1). We censored 22 (6%) individuals who initiated ART, 12 (4.1%) died before a diagnosis of virological failure (4 males and 8 females) while 10 (3.2%) were lost to follow up (4 males and 6 females). Twelve (4.1%) participants died after conventional virological failure and so were censored in the analysis of pragmatic failure. Participants who died were older than those who did not die (P = 0.05), were more immunosuppressed (P = 0.01) and more wasted (P = 0.01). Participants accrued 1035 person years at risk (pyar) and the median follow up time was 2.97 years (IQR), range 0.23 to 7.11 years. Five years after ART initiation, about 30% of the participants had developed conventional failure (Figure 2) and 10% of the participants had developed pragmatic virological failure (Figure 3).

**Table 1:** Participants’ characteristics at ART initiation

Factor	Categories	NumbersN	[Total=316](%)
Gender	Male	115	(36.4)
	Female	201	(63.6)
Age (Years)	14 – 29	67	(21.2)
	30 – 39	129	(40.8)
	40 – 49	79	(25.0)
	50+	41	(13.0)
Median age (IQR) in years	36.5 (IQR)		
Level of education	None or incomplete primary	213	(67.4)
	Complete primary/some secondary	48	(15.2)
	Complete secondary or higher	55	(17.4)
Marital status	Single, separated or widowed	139	(44.0)
	Married or with a steady partner	177	(56.0)
Number of steady partners at enrolment	None	135	(42.7)
	One	166	(52.5)
	Two or more	15	(4.75)
Occupation	Farmer	192	(60.8)
	Trader/seller	42	(13.3)
	Semi-skilled/skilled	18	(5.70)
	Others	64	(20.3)
Alcohol consumption	No	223	(70.6)
	Yes	93	(29.4)
CD4 Counts at ART initiation	Median (IQR)	*156*	*( 73 -200)*
CD4 group at ART initiation (cells/mm^3^)	<50	64	(20.3)
	50 - 199	173	(54.8)
	200+	79	(25.0)
^1^ Baseline viral load (copies per ml)	<400	15	(5.5)
	400 – 999	1	(0.4)
	1000-9999	21	(7.8)
	10000-99999	115	(42.3)
	100,000 – 999999	112	(41.2)
	1000000+	8	(2.9)
Viral load 1	Median (IQR 3)	81,758	(26,721 - 193,184)
^2^ Body mass index (BMI)(kg/m^2^)	<15	11	(3.5)
	15-18.5	74	(23.8)
	>18.5	226	(72.7)
Body mass index (2.9)	Mean(standard deviation)	*20.1*	

1 Baseline viral load has missing data on 44 participants

2 BMI has missing data for 5 participants , IQR - interquartile range

**Effect of adherence on conventional virological failure**: Overall, 84 (26.6%) participants developed conventional virological failure, a rate of 8.6 per 100 pyar. There were no significant differences in failure rates by gender, age and alcohol consumption. The median proportion of visits on which good adherence was reported was 97.0% (IQR (92-100). Conventional failure rates were significantly higher among participants who reported adherence less than 90% compared with participants who reported good adherence of above 95% of the visits (crude HR 1.38 for linear association with 10% drop in the proportion of visits with good adherence, 95% CI 1.23-1.55, P<0.001) (Table 2).

**Table 2:** Factors associated with conventional virological failure; crude failure rates and hazard ratios

Factor	Level	Number offailures N (pyar)	Failure ratePer 100 pyar	CrudeHazard ratios*(95% CI)	P-value^1^
Overall		8 (980)	8.6		
Gender	Male Female	28(359)	7.8	1	0.57
		5 (621)	9.0	1.14 (0.72- 1.80)	
Age in years	14 – 29	23(172)	13.4	1	0.28
	30 – 39	34 (436)	7.8	0.65 (0.38–1.10)	
	40 – 49	19 (262)	7.3	0.59 (0.32 – 1.10)	
	50+	8 (110)	7.3	0.57 (0.25 -1.27)	
Educational Level	None/incomplete Primary	54(647)	8.3	1	0.34
	incomplete secondary	16 (132)	12.1	1.43 (0 .82 - 2.50)	
	Complete secondary	14 (201)	7.0	0.85 (0.47 - 1.53)	
Marital Status	Single separated widowed	34 (452)	7.5	1	0.35
	Married/steady partner	50 (528)	9.5	1.22 (0.37 – 0.78)	
No of partners	None	34 (460)	7.4	1	0.32
	One	48 (480)	10.0	1.30 (0.25 –0.83)	
	Two or more	2 (41)	4.9	0.62 (0.15 -2.60)	
Consumes Alcohol	No	54 (683)	7.9	1	0.26
	Yes	30(298)	10.1	1.29 (0.83 -2.02)	0.99
Occupation	Farmer	51 (588)	8.7	1	
	Trader/seller	12 (146)	8.2	0.96 (0.51- 1.80)	
	Skilled / semi-skilled	4 (44)	9.1	0.95 (0.34- 2.62)	
	Other/missing	17 (202)	8.4	0.98 (0.56 – 1.71)	
CD4 at ART start Cells/ml	200+	17 (190)	8.9	1	0.62
	50- <200	46 (590)	7.8	1.02 (0.58-1.78)	
	< 50	21 (200)	10.5	1.30 (0.69–2.47)	
**^2^** Baseline viral loads (Copies/ml)	< 1000	2 (56)	3.6	1	0.62
	1000-<10,000	5 (57)	8.8	2.30 (0.45-11.88)	
	10000+	66 (835)	7.9	2.20 (0.56-9.28)	
Proportion of visits on which good adherence (>95%) was reported	95%+	47 (7)	6.7	1	<0.001
	90-94%	9 (1.6)	5.7	0.83 (0.41-1.71)	
	< 90%	28 (1.2)	23.1	2.99 (1.85-4.82)	
	Linear hazard ratio for 10% drop in the proportion of visits good adherence was reported			1.38 (1.231.55)
**^3^** Baseline Body mass index (BMI) Kg/m^2^	<18.5	57 (710)	8.0	1	0.68
	15-18.5	23 (233)	9.9	1.22 (0.76-1.99)	
	>15	2 (27)	7.4	0.85 (0.21-3.49)	

1 P value from the Likelihood Ratio Test

2 Baseline viral load has missing data on 11 participants

3 BMI has missing data for 2 participants

**Effect of adherence on pragmatic virological failure**: After the intensified ART adherence counselling, 28 (33.3%) of the 84 participants with conventional failure still had persistent VL > 1000 copies/ml, a pragmatic failure rate of 2.7 per 100 pyar. The pragmatic failure rate decreased significantly with increasing age (P = 0.04), but increased with decrease in proportion of visits on which good adherence was reported (P < 0.001) (Table 3). The unadjusted linear hazard ratio indicated that pragmatic failure rate increased by 45.0% for every 10% decrease in the proportion of visits on which good adherence was reported (HR 1.45, 95%CI 1.26-1.71; P < 0.001). After adjusting for age, gender, alcohol consumption, baseline CD4 counts and baseline VL, the adjusted linear hazard ratio for the failure rate per 10% decrease in the proportion of visits on which good adherence was reported increased to 2.12 (95%CI 1.61-2.79, P < 0.001).

**Table 3:** Factors associated with pragmatic virological failure; crude failure rates and hazard ratios

Factor	Category	No of failures N(pyar)	FailureRate / 100pyar	Hazard ratio (95% CI)	P-value^1^
Overall		28 (1036)	2.7		
Gender	Male	10 (384)	2.6	1	0.90
	Female	18 (652)	2.8	1.03 ( 0.52 –2.23)	
Age (Years)	14 – 29	12 (184)	6.5	1	0.04
	30 – 39	8 (463)	1.7	0.30 ( 0.09-0.71 )	
	40 – 49	5 (272)	1.8	0.31 ( 0.11-0.93 )	
	50+	3 (117)	2.6	0.43 ( 0.12-1.44 )	
Level of education	None or incomplete primary	19 (691)	2.7	1	0.33
	incomplete secondary	6 (138)	4.3	1.52 ( 0.62-3.92 )	
	Complete secondary	3 (207)	1.4	0.61 ( 0.24-1.56 )	
Marital status	Single, separated or widowed	11 (471)	2.3	1	0.33
	Married /steady partner	17 (564)	3.0	1.22 ( 0.64-2.67 )	
**^2^** Number of steady partners at enrolment	None	1 (481)	0.2	1	0.23
	One	17 (513)	3.3	1.34 ( 0.56-2.82 )	
	Two or more	0 ----	----	.	
Alcohol consumption	No	16 (717)	2.2	1	0.15
	Yes	12 (318)	3.8	1.67 ( 0.81-3.67 )	
Occupation	Farmer	17 (654)	2.6	1	0.15
	Trader/seller	1 (154)	0.6	0.33 ( 0.03-1.90 )	
	Semi-skilled/skilled	1 (47)	2.1	0.70 ( 0.11-5.30 )	
	Others	9 (211)	4.3	1.60 ( 0.74-3.67 )	
CD4 count at ART initiation (cells/mm^3^)	200+	7 (196)	3.6	1	0.19
	50 – <200	16 (625)	2.6	0.87 (0.36 – 2.12)	
	<50	5 (214)	2.3	0.78 (0.25 – 2.45)	
**^3^** Baseline viral load (copies per ml)	<1000	1 (58)	1.7	1	0.32
	1000- <10,000	2 (57)	3.5	1.91 ( 0.20-20.8 )	
	10000+	23 (886)	2.6	1.60 ( 0.20-11.7 )	
Proportion of visits on which good adherence (> 95%) was reported	95%+	12 (731)	1.6	1	<0.001
	90-94%	2 (162)	2.2	0.74 (0.16 – 3.29)	
	<90%	14 (139)	10.1	5.05 (2.3 –11.05 )	
	Linear hazard ratio for 10% drop in the proportion of visits good adherence was reported			1.45 (1.26 -1.71 )
**^4^** Body mass index (BMI)(kg/m^2^)	>18.5	16 (743)	2.2	1	0.33
	15-18.5	10 (255)	3.9	1.93 ( 0.87-4.25 )	
	>18.5	1 (28)	3.6	1.47 ( 0.20-11.11)	

1 P value from the Likelihood Ratio Test

2 Data was missing on the number of steady partners at enrolment for 10 participants with virological failure

3 aseline viral load has missing data on 2 participants

4 BMI has missing data^4^ for 1 participant

**Independent predictors of conventional virological failure**: After adjusting for age and gender, the only independent predictors of conventional virological failure were adherence and alcohol consumption. For each drop of 10% in the proportion of visits at which a participant achieved good adherence, the rate of failure increased by 83% (aHR 1.83, 95%CI 1.50-2.23, P < 0.001). Participants who consumed alcohol had 71% higher rate of conventional virological failure than those who reported not consuming alcohol (aHR 1.71, 95%CI 1.05-2.79, P = 0.03) (Table 4).

**Table 4:** Independent predictors of conventional virological failure-results from fitting cox’s models

Factor	Level	aHR (95% CI)	P-value^1^
Age in years	14 – 29	1	0.13
	30 – 39	0.70 (0.39 - 1.25)	
	40 – 49	0.62 (0.31 - 1.21)	
	50+	0.55(0.22 - 1.33)	
Proportion of adherent visits	Per 10% drop in proportion of visits on which adherence of 95% was reported	1.83 (1.50 - 2.23)	<0.001
Gender	Male	1	0.67
	Female	1.12 (0.67 - 1.87)	
Consumes alcohol	No	1	0.03
	Yes	1.71(1.05 - 2.79)	

aHR (95%CI) - adjusted Hazard ratio (95% confidence interval)

1 P-value from the likelihood ratio test

**Independent predictors of pragmatic failure among individuals with conventional failure**: We adjusted for apriori confounders; age, gender and alcohol consumption to determine predictors of pragmatic virological failure among participants with conventional virological failure. There was an 88% reduction in the odds of pragmatic failure from age group < 30 years to age group 30-39 years (adjusted odds ratio (aOR) 0.12, 95% CI 0.03-0.57, P = 0.01) and an 86% reduction in the odds of pragmatic failure from age group <30 years to age group 40 years and above (aOR = 0.14, 95%CI (0.03-0.71) P = 0.02). There was borderline evidence of a threefold increase in the odds of pragmatic failure among participants who reported consuming alcohol compared to those who reported not consuming alcohol (aOR = 3.14, 95% CI 0.95-10.34), P = 0.06). However, adherence was not significantly associated with pragmatic failure among participants with conventional failure (P = 0.44) (Table 5).

**Table 5:** Factors associated with pragmatic failure among individuals with conventional failure - results from logistic regression models

Factor	Level	ConventionFailuresNC=84	PragmaticFailuresNP=28	Proportion ofconventional failures who failed pragmatically (NP/NC) x 100	Adjusted odds ratios (95% CI)	P-value^1^
Age in years	14 – 29	23	12	(52.2%)	1	0.02
	30 – 39	34	8	(23.5%)	0.12 (0.03 – 0.57)	
	40 +	27	8	(29.6%)	0.14 (0.03 – 0.71)	
Proportion of visits adherence of > 95 was reported	95%+	47	12	(25.5%)	1	0.44
	<95%	37	16	(43.2%)	1.12 (0.84-1.50)**^2^**	
Consumes alcohol	No	54	16	(29.6%)	1	0.06
	Yes	30	12	(40.0%)	3.14 (0.95-10.34)	

1 P-value from the likelihood ratio test

2 Linear aOR showing the increase in failure rates for each 10% decrease in proportion of visits with good adherence

NC- number with conventional virological failure NP – number with pragmatic virological failure

## Discussion

In this rural Ugandan cohort, the pragmatic definition of virological failure resulted in a 66.7% reduction in the number of patients who would have been switched to second line ART based on the conventional virological failure definition. The independent predictors of conventional virological failure were ART adherence and alcohol consumption. Among individuals with conventional failure, the independent predictors of pragmatic failure were decreasing age and alcohol consumption. Our findings support the current ART guidelines recommendation to switch participants only after additional intensified ART adherence counselling and thus confirmation of true of pragmatic virological failure. This will help keep patients longer on first line ART regimen which is cheaper, safer and also preserves the more toxic and expensive second line ART regimen for as long as possible in a resource limited setting. Switching patients basing on conventional virological failure leads to higher proportions of patients getting switched unnecessarily. In a South African HIV cohort 21.6% of the patients were switched to second line due to detection of viraemia following ART initiation [28]. In our study, 26.6% of the 316 participants would have been switched to second line ART using the conventional failure definition, but when we used the pragmatic failure definition, only 8.9% were switched. The need to keep patients on first line ART regimens for as long as possible can be justified by their safety, lower cost and lesser pill burden [29]. Patients who fail on the lower pill burden and safer first line ART regimen have been shown to have a higher likelihood of virological failure after switching to second line ART regimen possibly due to poor adherence to a regimen with a higher pill burden and greater side effect profile [30]. The association between lower adherence and virological failure has been reported in other studies where adherence to drug refill was an early warning indicator to virological failure [28].

Decreasing age was the factor most significantly associated with pragmatic failure, however the small numbers younger patients made it impossible to explore this association in more detail, like finding the actual age below which failure is more likely. The higher pragmatic failure rates among participants aged below 30 years has been found in other studies. In Cameroon, a study on adults taking ART demonstrated that age independently predicted virological failure and an increase in age had better prediction in terms of immune, haematological and virological responses to ART [30]. In Nigeria, virological failure among younger participants was attributed to poor adherence [31] and poor adherence among participants aged 16-29 years was reported in Urban Zambia [32]. The social complexity of reasons for ART non-adherence among adolescents and young people have also been reported [30]. However, the biological reasons for high virological failure rates among younger adults need to be understood. In our study, alcohol consumption was an independent risk factor for conventional failure and was also associated with pragmatic failure, although the association was of borderline statistical significance. Previous studies have shown that alcohol affects adherence through a complex mechanism and a significant association between alcohol use and poor ART adherence has been demonstrated before [33-35]. Alcohol might also worsen tolerability of ART agents which may lead to deliberate skipping of doses to avoid the side effects. Reports from a prospective cohort showed that a substantial number of people intentionally skip or stop their ART medications when drinking alcohol [33]. Most of our study participants were females, other studies on ART in SSA have also reported a female dominance among participants, in South Africa (67.8%), in Cameroon (77.8%) and in Tanzania (64.1%) most of the patients on ART were females [27, 36, 37]. Most of our participants were aged below 40 years, this is also similar to what has been found in other African settings [30]. The low median CD4 count at ART initiation of 156 cells/mm3 is due to the fact that by the time of universal roll out of ART, many PLWHA were already severely immunosuppressed, a finding that is common sub-Saharan Africa where ART is a relatively recent intervention [38].

**Strengths**: The prospective study design enabled consistent follow up of participants for a median period of about 3 to 7 years which allowed assessment of the temporal association between virological failure and the exposures. This follow up also enhanced strict intensive adherence counselling for 6 months among patients with conventional virological failure before detecting pragmatic failure. Death, migration and missed appointments were well recorded and loss to follow up was low at 3.2%, which minimised the likelihood of selection bias. The few participants with missing baseline data were comparable to those without missing data. Regular viral loads measurement reduced the likelihood of misclassification of outcome. Participants were recruited from a general population based cohort minimising selection bias and enhancing generalizability of findings. The study had a robust data collection mechanism, with trained staff and laboratory equipment were subjected to regular external validity checks and quality assurance to minimise errors in the measurement of viral load and CD4 count.

**Limitations**: Our estimates were subjected to random error by having few participants aged below 30 years and above 50 years. This undermined exploration of the age effect like to find the actual age below or above which failure is more or less likely. Few participants had pragmatic virological failure which might have led to imprecise estimates and low power to detect associations between risk factors and outcome. Participants who died differed significantly from those who did not die and were likely to be older, have lower BMI and were more immunosuppressed. Treating deaths as censored observations might have biased the estimates of virological failure especially if those who died were more likely to have developed virological failure. The measured self-reported alcohol consumption was prone to social desirability bias, and the quantity, type and frequency of alcohol consumption was not recorded. The likely recall bias arising from self-reported ART adherence was minimised by asking participants if they skipped any doses in the last 4 days, however, the analysis of adherence as a summary variable ignored the temporal variation of adherence. We assessed a limited number of potential confounders so virological failure estimates might be subjected to residual confounding. We did not establish mortality or test ART drug resistance mutations within the 6 months participants with virological failure were subjected to strict adherence counselling, which would have been important in evaluating the safety of the pragmatic failure approach.

## Conclusion

Our findings reinforce the need to sustain viral load testing as a key tool in monitoring treatment progress and to use the safe and cheap pragmatic approach when switching participants from first to second line ART in resource limited settings to preserve second line ART regimen. To achieve the 90-90-90 treatment target by 2020 in SSA, the strategy of rolling out viral load testing should be supplemented with innovative adherence enforcement to achieve sustained viral suppression among patients on ART. Maintaining adequate stocks of ART and patient tailored counselling especially for the young and alcohol users will prevent poor adherence and virological failure. National governments should increase funding for recruiting or training staff in ART adherence counselling. Qualitative studies are however needed to assess the factors responsible for virological failure among the alcohol consumers and young people as this will help design strategies to address adherence issues and virological failure in these groups.

### What is known about this topic

- WHO guidelines currently recommend viral load testing as the gold standard for monitoring and confirming treatment response;
- WHO also recommends Intensified adherence counselling following an initial high viral load (> 1000 copies/mL) before conducting a second viral load test;
- Adherence is a known independent predictor of virological failure.

### What this study adds

- The study demonstrates the independent predictors for both pragmatic and conventional failures including the young age and alcohol consumption in African populations which will direct policy on the need to design strategies to deal with virological failure focusing on these categories;
- The study also presents a basis for qualitative exploration of Virological failure among young people and alcohol consumers;
- The study results show a significant (66.7%) reduction in the number of participants unnecessarily switched to second line ART if the conventional VF approach was used alone which further strengthens the importance of using a pragmatic virological failure approach in switching to second line ART.
